# LncRNA RP11-138J23.1 Contributes to Gastric Cancer Progression by Interacting With RNA-Binding Protein HuR

**DOI:** 10.3389/fonc.2022.848406

**Published:** 2022-03-22

**Authors:** Yongcan Xu, Xiang Yu, Jing Xu, Jun Lu, Hao Jiang, Neng Lou, Wei Lu, Jiewei Xu, Guochao Ye, Shunli Dong, Fengqi Nie

**Affiliations:** ^1^ Department of General Surgery, Huzhou Central Hospital, Affiliated Central Hospital, Huzhou University, Huzhou, China; ^2^ Department of General Surgery, The Affiliated Yantai Yuhuangding Hospital of Qingdao University, Yanta, China; ^3^ Department of Neurology, The Second Affiliated Hospital of Nanjing Medical University, Nanjing, China; ^4^ Department of Central Laboratory, Huzhou Central Hospital, Affiliated Central Hospital, Huzhou University, Huzhou, China; ^5^ Department of Oncology, Second Affiliated Hospital, Nanjing Medical University, Nanjing, China

**Keywords:** lncRNA, RP11-138J23.1, gastric cancer, HuR, mRNA stability

## Abstract

In spite of improvements in diagnostics and treatment of gastric cancer (GC), it remains the most common malignancy of human digestive system. It is now widely appreciated that long noncoding RNAs (lncRNAs) exert extensive regulatory effects on a spectrum of fundamental biological processes through diverse mechanisms. In this study, we explored the expression level and functional role of lncRNA RP11-138J23.1 in GC. Through bioinformatics analyses and *in situ* hybridization (ISH), we identified that RP11-138J23.1 was upregulated in GC tissue. Further study showed that RP11-138J23.1 knockdown significantly inhibited cell proliferation and metastatic ability. Whereas, RP11-138J23.1 overexpression could promote tumor cell growth and metastasis *in vitro*. Additionally, loss-of-function assays were used to confirm the role of RP11-138J23.1 *in vivo*. Mechanistically, RP11-138J23.1 exerted its oncogenic functions by binding to HuR protein and increasing stability of VAV3 mRNA. Overall, our study highlights the essential role of RP11-138J23.1 in GC, suggesting that RP11-138J23.1 might be a potent therapeutic target for patients with GC.

## Introduction

Gastric cancer (GC) characterized with malignant cell growth and metastasis is the most common malignancy of the human digestive system (1). High incidence rate of GC is observed in China, which is ranked second in cancer (2). The majority of patients suffering from GC are diagnosed at advanced stages since the symptoms of early gastric cancer are not obvious. Although the overall survival (OS) rate of GC has been improved due to advanced novel therapeutic approaches (3), the overall 5-year relative survival rate remains very poor (4). The dominant reason for such a high mortality rate is to maintain viability and metastatic potential. Hence, further exploration of novel biomarkers and therapeutic targets for GC diagnosis and treatment is urgent.

Compared with protein-coding genes, long noncoding RNAs (lncRNAs) are characterized with limited or no protein-coding capacity (5). In recent years, lncRNAs have drawn extensive attention with the progression of whole genome and transcriptome sequencing technologies (6). Indeed, further evidence indicated that lncRNAs exert essential functions in physiological and pathological processes, such as cell differentiation, metabolism, and carcinogenesis (7–9). Recent studies have reported that lncRNA could increase oncogene or decrease tumor suppressor genes through lncRNA-protein interaction or lncRNA-miRNA interaction (10, 11). Therefore, dysregulated lncRNAs might be potential biomarkers and targets in different type of human cancers. Our previous studies confirmed that lncRNA DUXAP10 promotes GC carcinogenesis by regulating LATS1 and β-catenin (12). In addition to DUXAP10, Xie et al. found that LINC00707 was upregulated in GC and facilitated cell proliferation and metastasis *via* interacting with mRNA-stabilizing protein HuR (13). In this regard, more specific mechanisms of lncRNAs in the initiation and progress of GC need to be further teased out.

The focus of this study was profiling the novel candidate lncRNAs responsible for the progression of GC and identified a gastric cancer-associated lncRNA RP11-138J23.1 (chr5:104079911-104105403) *via* bioinformatics analysis. We further explored the functional roles and molecular mechanism of RP11-138J23.1 in GC progression.

## Materials and Methods

### Gene Expression Datasets

The RNA sequencing data of The Cancer Genome Atlas (TCGA) GC tissues and the corresponding clinical data were downloaded from TCGA database. Another three public GC RNA sequencing and microarray datasets (GSE54129, GSE79973, and GSE99416) were downloaded from the Gene Expression Omnibus (GEO). In additional, the scores between RP11-138J23.1 or VAV3 mRNA and HuR protein were predicted *via* RNA-Protein Interaction Prediction website (http://pridb.gdcb.iastate.edu/RPISeq/).

### Tissue Microarray (TMA) and RNA Fluorescent *In Situ* Hybridization

We obtained tissue microarray (TMA) containing 74 pairs of GC and normal gastric tissues from Outdo Biotech (Shanghai, China). RNA ISH was performed to detect RP11-138J23.1 expression in TMA using probe (BOSTER Biological Technology Co. Ltd., Wuhan, China). After dewaxing and rehydration through different concentrations of ethanol solutions, the TMA were digested with proteinase K at 37°C and hybridized with the digoxin-labeled probe 3–6 h at 55°C.

### Cell Culture and Transfection

Human gastric normal epithelium cell line (GES-1) and GC cells (BGC-823, SGC-7901, and MGC-803) were purchased from Shanghai Institutes for Biological Science, China. BGC-823, SGC-7901 and MGC-803 cells were cultured in RPMI 1640 medium (KeyGene, Nanjing, China). GES-1 cells were cultured in DMEM medium (KeyGene, Nanjing, China) supplemented with 1% penicillin/streptomycin, l-glutamine, and 10% fetal bovine serum (FBS, Invitrogen, Carlsbad, CA, USA). Culture plates were incubated at 37°C in a humidified atmosphere with 5% CO^2^.

The siRNA specifically targeting RP11-138J23.1, HuR and VAV3 was designed and synthesized by Invitrogen (Invitrogen, USA). The nucleotide sequences of all siRNAs were shown in **Supplementary Table S1**. The cells were transfected with siRNA using RNAiMAX (Invitrogen, USA). In addition, RP11-138J23.1 overexpression plasmid and vectors containing shRNA targeting RP11-138J23.1 was constructed and transfected with XtremeGENE HP DNA transfection reagent (Roche, Basel, Switzerland).

### RNA Isolation and Quantitative Reverse Transcription-Polymerase Chain Reaction Analyses

Total RNA was extracted from cells or tissue samples by TRIzol reagent (Invitrogen, Carlsbad). The RNA quality was evaluated by Nanodrop ND-2000 spectrophotometer. According to the manufacturer’s instructions, 1 µg RNA was reverse transcribed into cDNA with the PrimeScript RT Reagent Kit (TaKaRa, Dalian, China). For detection of lncRNA and mRNA, quantitative reverse transcription-polymerase chain reaction (qRT-PCR) was performed using the SYBR Premix Ex Taq II (Perfect Real Time) on a 7500 Fast Real-Time PCR System (Applied Biosystems, Foster City, CA, USA). The expression of β-actin was used for normalization. Related primers are listed in **Supplementary Table S1**.

### MTT Assay and Colony-Formation Assay

MTT assay was conducted for *in vitro* proliferation assay in accordance with the manufacturer’s instructions. GC cells, transfected with siRNA, scrambled siRNA, pcDNA3.1, or pcDNA3.1-RP11-138J23.1 vector for 48 h, were plated at a density of 1,500 cells/well in 96-well plates. Cell viability was documented every 24 h. The experiment was performed with three replicates, and six parallel samples were performed each time.

For colony-formation assay, a total of 700 transfected cells were suspended in a fresh six-well plate and maintained in media containing 10% FBS. After the formation of the colony, the plate was gently washed with PBS and fixed with methanol for 10 min, and then stained with 0.1% crystal violet (Sigma-Aldrich, America). Visible colonies more than 50 cells were manually counted. Triplicate wells were assessed for each treatment group.

### Flow Cytometric Analysis of Apoptosis and Cell Cycle

Flow-cytometric analysis was carried out to evaluate cell apoptosis and cell cycle. Forty-eight hours after transfection, cells for apoptosis analysis were stained with propidium iodide (PI) and FITC-Annexin V following the protocol of the FITC Annexin V Apoptosis Detection Kit (BD Biosciences). The cells were then analyzed by FACScan. Cells were divided into viable cells, early apoptotic cells, late apoptotic cells, and dead cells. The ratio of early apoptotic cells and late apoptotic cells was measured.

For the flow-cytometric analysis of the cell cycle, the transfected cells were fixed in 75% ice-cold ethanol at −20°C overnight. Cells were then stained with propidium oxide by the CycleTEST PLUS DNA Reagent Kit (BD Biosciences). The percentage of G0/G1, S, and G2/M phases was analyzed with a flow cytometry (FACScan^®^; BD Biosciences).

### Transwell Assay

To determine the ability of migration and invasion, Transwell assays were conducted. For cell migration, 5 × 10^4^ cells were seeded into the upper chamber of Transwell assay (Millipore, Billerica, MA, USA) containing 200 μl of serum-free medium with a membrane (5 μm pores). Medium containing 10% FBS was filled into the lower chamber. For invasion assays, 1 × 10^5^ transfected cells were seeded into the upper chamber with Matrigel and the lower chamber were filled with medium containing 10% FBS. After 24 h, the cells on the lower membrane surface were fixed with 4% paraformaldehyde, stained with crystal violet, and photographed with a digital microscope. Finally, the cell numbers were imaged and counted in five random fields for each chamber.

### Xenografts

Male BALB/c nude mice (4-week-old) were used for the tumor formation assay. Animal experiments were approved by the Institutional Animal Care and Use Committee of Zhejiang University (Hangzhou, China). BGC-823 cells transfected with shRNA targeting RP11-138J23.1 or empty vector were harvested and resuspended at a concentration of 1 × 10^7^ cells/ml. A total of 100 µl of suspended cells was subcutaneously injected into a single flank of each mouse (*n* = 6). Tumor growth was measured using calipers every week, and growth curves were plotted for each group. Tumor volumes were calculated using the equation: *V* = 0.5 × *D* × *d* ^2^ (*V*, volume; *D*, longitudinal diameter; *d*, latitudinal diameter). Tumors were removed from all animals after 4 weeks, and tumor weights were measured. Parts of the tumors were further detected by the expression of Ki-67 *via* immunohistochemistry and RP11-138J23.1 by qRT-PCR.

### Western Blot Analysis

The GC cells was lysed using lysis buffer containing the mammalian protein extraction reagents RIPA (Beyotime China), protease inhibitor cocktail (Roche, Basel, Switzerland), and PMSF (Roche). Followed by centrifugation at 13,000 rpm for 20 min, the concentration of total protein was determined using the Pierce™ BCA Protein Assay Kit (Thermo Scientific™, USA). Fifty micrograms of the protein lysates were electrophoresed on 10% SDS-polyacrylamide gel electrophoresis (SDS-PAGE), transferred to 0.22 μm NC membranes (Sigma), and incubated with specific antibodies. The specific bands were visualized using an ECL detection reagent (BioRad, USA) and quantified by densitometry (Quantity One software, BioRad). VAV3 antibody was purchased from Abcam (Cambridge, USA), and β-actin (Abcam) was used as a control.

### Subcellular Fractionation Location

Nuclear and cytosolic fractions were separated and purified using the PARIS Kit (Life Technologies, Carlsbad, CA, USA) according to the manufacturer’s instructions.

### RNA Immunoprecipitation Assays

A Magna RIP RNA-Binding Protein Immunoprecipitation Kit (Millipore, Billerica, MA, USA) was used for RNA immunoprecipitation (RIP) experiments following the manufacturer’s instructions. BGC-823 cells were lysed, and the cell extract was incubated with magnetic beads conjugated with anti-HuR and anti-IgG at 4°C. Immunoprecipitated RNA was purified and analyzed *via* qRT-PCR. Antibodies for RIP assays against HuR and IgG were purchased from Millipore.

### RNA Stability Assays

GC cells were transfected with siRNAs or overexpression vectors for 48 h, and then treated with actinomycin D (1 μg/ml). For further determination of RNA and protein, cells were collected at different time points.

### Statistical Analysis

SPSS19.0 software (IBM, Chicago, IL, USA) was used for all statistical analyses, and differences were considered to be statistically significant with *p* < 0.05. The Student’s *t*-test (two-tailed) and Chi-square test were used to analyze differences between groups. All data were presented as the mean ± SD.

## Results

### RP11-138J23.1 Is Highly Expressed in Human GC Tissues

**Figure 1:**
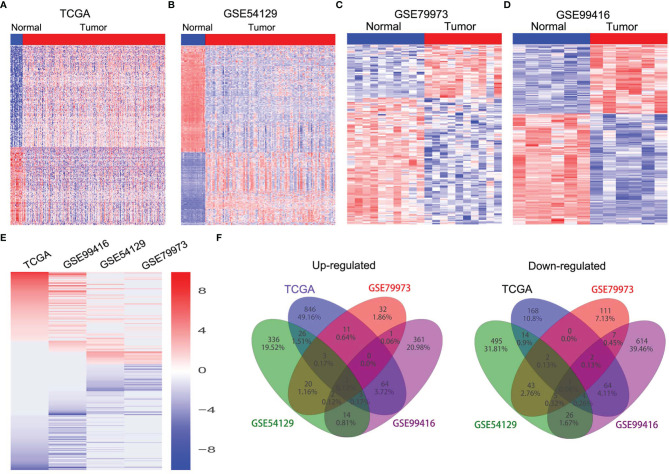
The expression profiles of lncRNAs in GC tissues. **(A)** Heatmap of the differentially expressed lncRNA expression in GC and normal tissue samples was analyzed using the TCGA datasets. **(B)** Heatmap of the differentially expressed lncRNA expression in GC and normal tissue samples was analyzed using the GSE54129 datasets. **(C)** Heatmap of the differentially expressed lncRNA expression in GC and normal tissue samples was analyzed using the GSE79973 datasets. **(D)** Heatmap of the differentially expressed lncRNA expression in GC and normal tissue samples was analyzed using the GSE99416 datasets. **(E)** Heatmap of the altered lncRNAs profiling in TCGA, GSE54129, GSE79973, GSE99416 datasets. **(F)** Venn diagram of altered lncRNAs in TCGA, GSE54129, GSE79973, and GSE99416 datasets.

**Figure 2:**
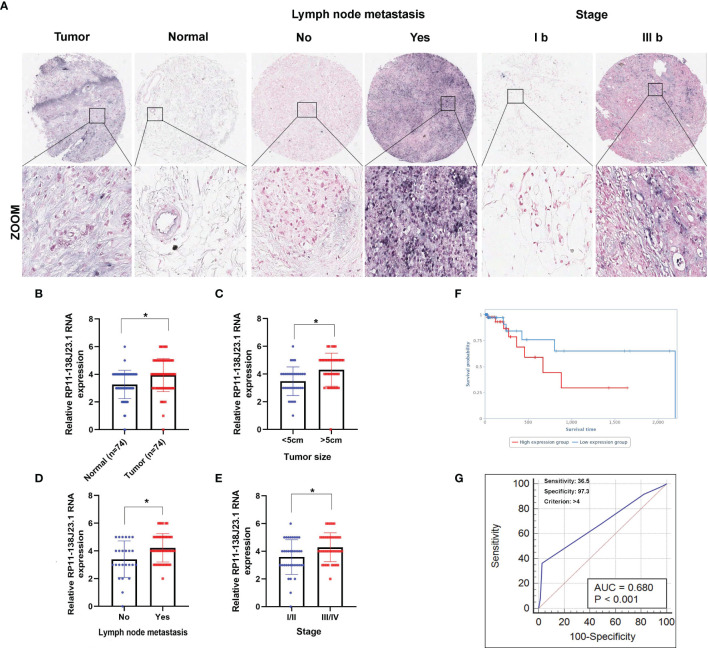
Higher RP11-138J23.1 expression levels in GC and its clinical significance. **(A)** The RP11-138J23.1 expression level in 74 GC tissues and corresponding adjacent nontumor tissues was quantified by ISH analysis. **(B)** Histogram showing RP11-138J23.1 *in situ* hybridized expression of paired adjacent nontumor tissue and GC tumors. **(C–E)** The relationship between RP11-138J23.1 expression and clinicopathological parameters (such as maximum diameter, lymphatic metastasis, and TNM stage) was shown. **(F)** Kaplan-Meier survival plots demonstrated that higher RP11-138J23.1 abundance correlated with a poor OS using the TCGA datasets. **(G)** The ROC curve for prediction of GC based on the expression level of RP11-138J23.1, using paired adjacent nontumorous tissues as a control. ^*^ *p* < 0.05.

In order to identify dysregulated lncRNAs in human GC, we firstly downloaded and analyzed GC microarray gene-profile datasets from GEO (GSE99416, GSE54129, and GSE79973) and RNA sequencing datasets from TCGA (**Figures 1A–D**). As presented in **Figures 1E, F**, four datasets shared 2 and 1 lncRNAs that were upregulated and downregulated, with a ≥2.0-fold change relative to their corresponding normal counterparts. To elucidate the diagnostic, therapeutic, and prognostic roles of lncRNAs in GC, RP11-138J23.1, significantly increased in all four datasets, was chosen for further analysis. To confirm the reliability of the above bioinformatics analysis results, the level of RP11-138J23.1 was ascertained by *in situ* hybridization (ISH) in 74 paired clinical GC tissues and corresponding normal gastric tissues. We found that RP11-138J23.1 is mainly located in the cytoplasm and the positive lesions are manifested as red. The result confirmed the aberrant upregulation of RP11-138J23.1 in GC tissue compared with adjacent normal tissues (**Figures 2A, B**). Subsequently, survival analysis was performed in 285 stomach adenocarcinoma samples by using the bioinformatics tool “TANRIC”. As a result, high expression level of RP11-138J23.1 indicated a poorer prognosis in stomach adenocarcinoma patients (Cox *p*-value = 0.041) (**Figure 2F**). Overall, these findings revealed that RP11-138J23.1 might exert a crucial role in GC development.

### Upregulation of RP11-138J23.1 Is Correlated With GC Malignant Progression

To explore the clinical significance of RP11-138J23.1, we evaluated the correlation of RP11-138J23.1 expression level with clinicopathological parameters (i.e., tumor size, lymphatic metastasis, or TNM stage). According to the median value of RP11-138J23.1, 74 patients were further classified into high- and low-level groups. Highly expressed RP11-138J23.1 was found to exhibit a significant correlation with larger tumors, lymph node metastasis, and advanced stage (*p* < 0.05, **Figures 2C–E** and **Table 1**).

**Table 1:** Association of RP11-138J23.1 level with clinicopathological parameters of patients with GC.

Variables	N of cases (%)	RP11-138J23.1 expression level		
		Low	High	*p*-value
		*n* = 26	*n* = 48	
Age (year, mean = 62.5)				
<62.5	33 (45%)	11	22	0.77
≥62.5	41 (55%)	15	26	
Gender				
Men	57 (77%)	20	37	1
Women	17 (23%)	6	11	
Differentiation				
Well, moderate	23 (31%)	10	13	0.312
Poor	51 (69%)	16	35	
Tumor size (maximum diameter (cm), mean = 5 cm)				
<5 cm	33 (45%)	16	17	0.031
≥5 cm	41 (55%)	10	31	
Distant metastasis				
No	66 (89%)	25	41	0.1
Yes	8 (11%)	1	7	
Lymph node metastasis				
Positive	49 (66%)	13	36	0.03
Negative	25 (34%)	13	12	
TNM stage				
I/II	36 (49%)	17	19	0.034
III/IV	38 (51%)	9	29	

Additionally, using corresponding normal gastric tissues as control, we plotted a receiver operating characteristic (ROC) curve to investigate the diagnostic value of RP11-138J23.1 diagnosing GC. The cutoff value to differentiate the benign and malignant gastric tissues was 4 (ISH score). The specificity and sensitivity were 97.3% and 36.5%, respectively, and the AUC of the ROC curve was 0.68 (**Figure 2G**). These data demonstrated that RP11-138J23.1 may act as a diagnostic biomarker for human GC because of the high specificity.

### RP11-138J23.1 Regulates GC Cell Proliferation *In Vitro*

**Figure 3:**
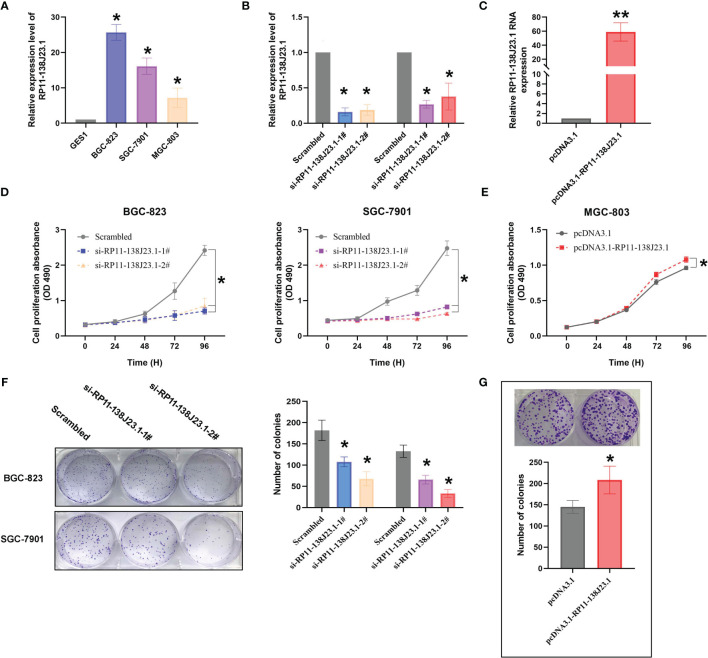
The effects of RP11-138J23.1 on GC cell viability *in vitro*. **(A)** qRT-PCR analysis was performed to identify the expression level of RP11-138J23.1 in GC cell lines and human gastric mucosa cell (GES1). The expression levels are normalized to GES1. **(B)** The RP11-138J23.1 expression level in BGC-823 and SGC-7901 transfect with two discrete chemically synthesized siRNAs. **(C)** The RP11-138J23.1 expression level in MGC-803 transfected with pcDNA3.1 RP11-138J23.1 and empty vector. **(D)** MTT assays were used to measure the growth curve of si-RP11-138J23.1-transfected BGC-823 and SGC-7901 cells. **(E)** MTT assays were used to measure the growth curve of pcDNA3.1 RP11-138J23.1 and empty vector transfected in MGC-803. **(F)** Colony-forming assays were conducted to determine the proliferation of si-RP11-138J23.1-transfected BGC-823 and SGC-7901 cells. **(G)** Colony-forming assays were conducted to determine the proliferation of pcDNA3.1 RP11-138J23.1 and empty vector transfected in MGC-803. ^*^ *p* < 0.05; ^**^ *p* < 0.01. Values indicate the mean ± SD from three independent experiments.

The RNA expression levels of RP11-138J23.1 in the human GC cell lines (BGC-823, SGC-7901, MGC-803) and the normal gastric mucosa cell line (GES-1) were examined by RT-qPCR. Compared with GES-1, RP11-138J23.1 is shown as obviously more abundant in GC cell lines (**Figure 3A**). To explore the biological functions of RP11-138J23.1 in GS, we used two siRNAs to downregulate endogenous RP11-138J23.1 level in BGC-823 and SGC-7901 cells. Forty-eight hours posttransfection, RP11-138J23.1 expression levels were knocked down by more than 60% (**Figure 3B**). Meanwhile, RP11-138J23.1 was ectopically expressed with pcDNA3.1-RP11-138J23.1 vector in MGC-803 cells and the efficiency of transfection was 58.9-fold (**Figure 3C**).

When RP11-138J23.1 was silenced, cell viability assessed by MTT assays in BGC-823 and SGC-7901 cells decreased (**Figure 3D**). Consistently, the cell viability of MGC-803 in the pcDNA3.1-RP11-138J23.1 group exceeded that of the pcDNA3.1 group (**Figure 3E**). Colony-formation assay was conducted to evaluate the clonogenic survival potential of cells, and we obtained similar results (**Figures 3F, G**).

**Figure 4:**
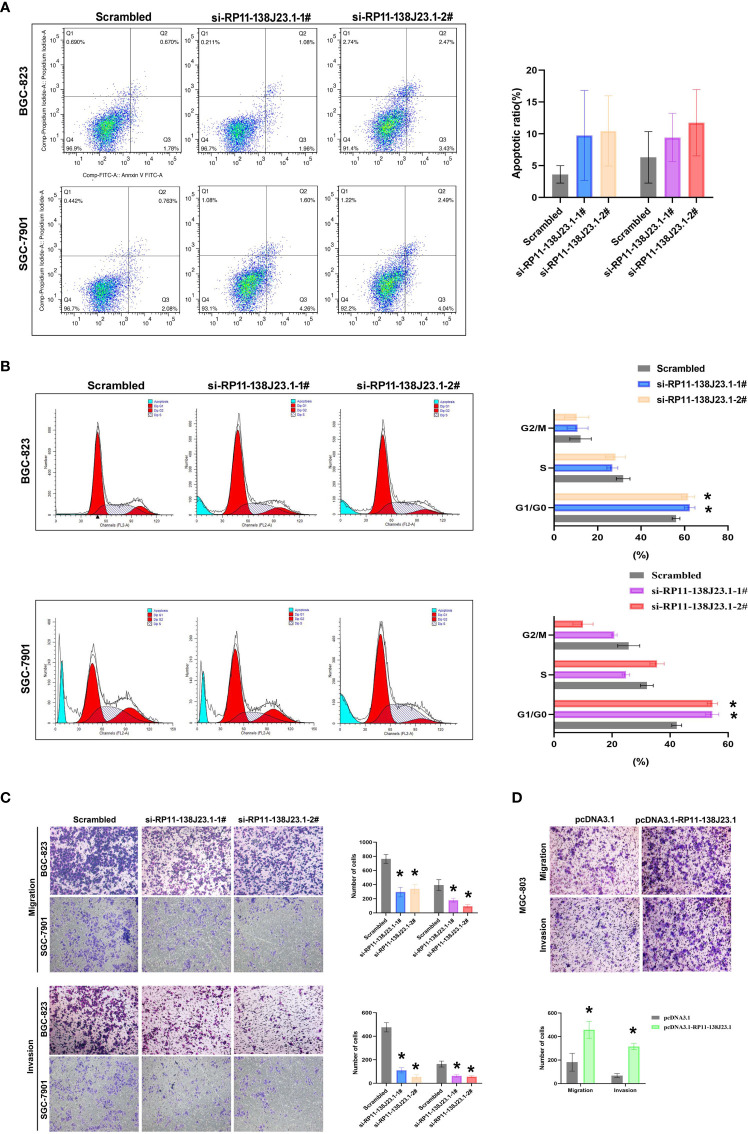
The effects of RP11-138J23.1 on GC cell apoptosis, cell cycle, cell migration, and invasion *in vitro*. **(A)** The apoptosis of BGC-823 and SGC-7901 were analyzed by flow cytometry. LR, early apoptotic cells; UR, terminal apoptotic cells. **(B)** Flow cytometry assays were performed to analyze the cell cycle progression when GC cells were transfected with RP11-138J23.1. **(C)** Transwell assays were used to determine the cell metastatic ability of si-RP11-138J23.1 1#, si-RP11-138J23.1 2#, or scrambled transfected BGC-823 and SGC-7901 cells. The cells on the lower chamber were stained and presented. **(D)** Transwell assays were used to determine the cell metastatic ability of pcDNA3.1 RP11-138J23.1 and empty-vector-transfected MGC-803 cells. Data represent the mean ± SD from three independent experiments. ^*^ *p* < 0.05. NS, not significant.

To further determine the impact of RP11-138J23.1 on GC cell growth *via* affecting cell cycle or apoptosis, FACS analysis was conducted. Suppression of RP11-138J23.1 led to an increase in the number of cells in the G0/G1-phase (**Figure 4B**). In addition, we also found that depletion of RP11-138J23.1 elevated seemingly higher levels of apoptosis in GC cells (**Figure 4A**). Unfortunately, it was not statistically significant.

### Downregulation of RP11-138J23.1 Reduces Migration and Invasion of GC Cells

In cancer malignant progression process, metastasis was an important biological feature. The results of Transwell assay demonstrated that RP11-138J23.1 knockdown inhibited the migratory and invasion capability of BGC-823 and SGC-7901 cells, while the capability of migratory and invasion observably increased once RP11-138J23.1 was overexpressed (**Figures 4C, D**). Together, these data indicated that RP11-138J23.1 plays an important role in GC metastasis.

### RP11-138J23.1 Promotes GC Cells Tumor Growth *In Vivo*

**Figure 5:**
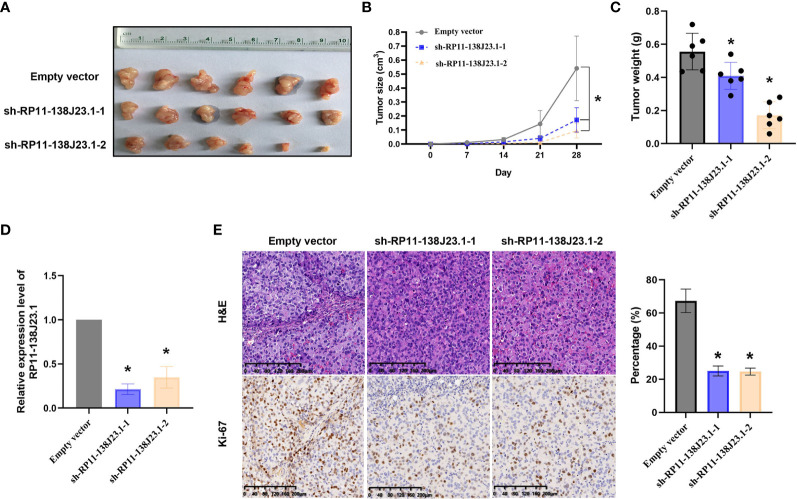
The effects on tumor growth after RP11-138J23.1 downregulation *in vivo*. **(A, B)** The tumor growth curves were measured 7 days after the injection of BGC-823 cells stably transfected with shRNA-RP11-138J23.1 1#, shRNA-RP11-138J23.1 2#, or empty vector, and tumor volume was calculated once every 7 days. **(C)** Tumor weight when the tumors were harvested. **(D)** qRT-PCR analysis of RP11-138J23.1 expression level in tumor tissues formed from shRNA-RP11-138J23.1 1#, shRNA-RP11-138J23.1 2#, or empty-vector-transfected BGC-823 cells. **(E)** Representative images of HE staining and Ki-67 immunohistochemistry of the tumor. up, H & E staining; down, immunostaining. The data represent the mean ± SD from three independent experiments. ^*^ *p* < 0.05.

To further estimate the oncogenic role of RP11-138J23.1 *in vivo*, we employed a mouse xenograft model. We established stable knockdown BGC-823 cells through a lentiviral vector harboring a short hairpin RNA targeting RP11-138J23.1 (sh-RP11-138J23.1-1 and sh-RP11-138J23.1-2). Cells stably transfected with either sh-RP11-138J23.1 or control empty vector were subcutaneously injected into nude mice. Indeed, tumors derived from sh-RP11-138J23.1-1 and sh-RP11-138J23.1-2 cells had significantly smaller tumor volumes and lower tumor weights compared with control empty vector mice (**Figures 5A–C**). The expression of RP11-138J23.1 was confirmed down-regulated in sh-RP11-138J23.1-1 and sh-RP11-138J23.1-2 group by qPCR analysis (**Figure 5D**). Meanwhile, compared with the control tumors, sh-RP11-138J23.1-1 and sh-RP11-138J23.1-2- derived tumors exhibited lower levels of the proliferation marker Ki-67 (**Figure 5E**).

### RP11-138J23.1 May Be Involved in GC Biology by Interacting With RNA-Binding Protein HuR

**Figure 6:**
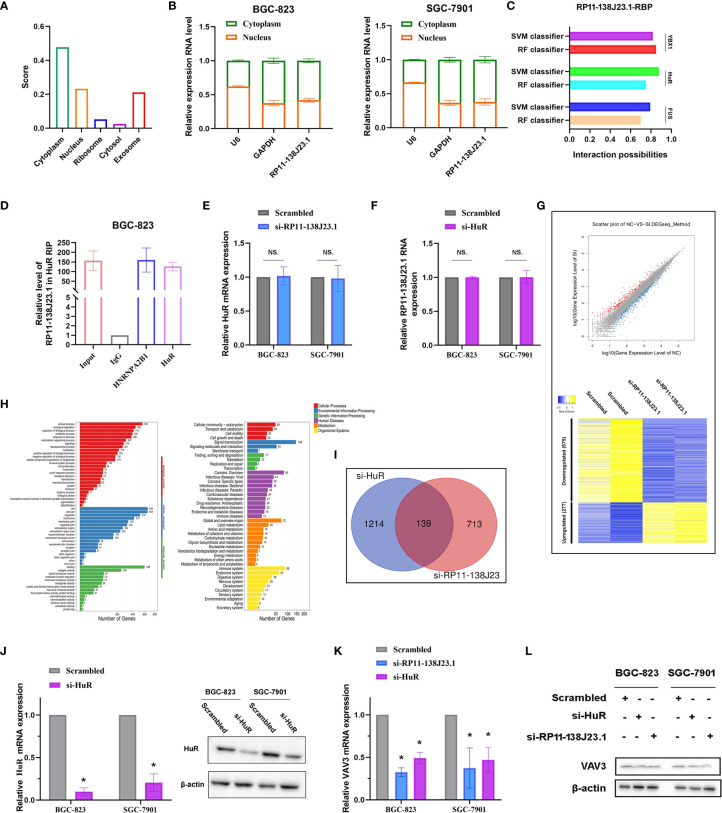
RP11-138J23.1 regulated VAV3 expression level *via* interacting with HuR. **(A)** The subcellular localization of RP11-138J23.1 *via* prediction website (http://www.csbio.sjtu.edu.cn/bioinf/lncLocator/). **(B)** RP11-138J23.1 expression levels in different subcellular fractions in BGC-823 and SGC-7901. U6 was used as a nucleus marker, and GAPDH was a cytosol marker. **(C)** The interaction probabilities between HuR and RP11-138J23.1 were predicted by the RNA-Protein Interaction Prediction (RPISeq) website. **(D)** RIP with rabbit monoclonal anti-HuR and preimmune IgG from BGC-823 cell extracts. RNA levels in immunoprecipitates were detected by qPCR. Expression levels of RP11-138J23.1 RNA are presented as fold enrichment in HuR relative to IgG immunoprecipitates. **(E)** qRT-PCR analysis of HuR mRNA expression level in BGC-823 and SGC-7901 after RP11-138J23.1 knockdown. **(F)** qRT-PCR analysis of RP11-138J23.1 RNA expression level in BGC-823 and SGC-7901 after HuR knockdown. **(G)** RNA transcriptome sequencing analysis was performed to analyze gene expression profiling in BGC-823 cells following RP11-138J23.1 knockdown. Volcano plot and heatmap showed all the differently expressed genes. **(H)** Go cellular component and KEGG pathway analysis for all genes with altered expressions between the negative control group and RP11-138J23.1 knockdown group cells *in vitro*. **(I)** In total, 139 transcripts were altered simultaneously in si-RP11-138J23.1-treated cells and si-HuR-treated cells (fold change >2, *p* < 0.05). **(J)** The HuR mRNA and protein expression level in BGC-823 and SGC-7901 transfect with siRNAs. **(K)** The VAV3 mRNA expression level in BGC-823 and SGC-7901 transfect with si-RP11-138J23.1 and si-HuR. **(L)** The VAV3 protein expression level in BGC-823 and SGC-7901 transfect with si-RP11-138J23.1 and si-HuR. The data represent the mean ± SD from three independent experiments. ^*^ *p* < 0.05. NS, Not Significant.

Based on our preliminary evidence, we highlighted an important role of RP11-138J23.1 in human GC; however, the mechanism(s) governing the oncogenic role of RP11-138J23.1 in GC have yet to be elucidated. Knowledge of subcellular localization of lncRNAs might provide a clue about lncRNA mechanisms. RP11-138J23.1 was proved to be predominantly located in the cytoplasm *via* the prediction website (http://www.csbio.sjtu.edu.cn/bioinf/lncLocator/) (**Figure 6A**) and confirmed in GC tissues using ISH. Coincidentally, we performed a subcellular fractionation assay and determined that RP11-138J23.1 transcript is enriched in the cytoplasmic fraction in GC cells (**Figure 6B**). These data strongly support that RP11-138J23.1 has distinct cytoplasm localization and likely participates in posttranscriptional regulation.

To further investigate the underlying mechanisms of RP11-138J23.1, we predicted the interaction probabilities of RP11-138J23.1 and RNA-binding protein *via* the RNA-Protein Interaction Prediction website (http://pridb.gdcb.iastate.edu/RPISeq/). As presented in **Figure 6C**, the interaction probabilities of RP11-138J23.1 with FUS, HuR, and YBX1 were 0.7, 0.75, and 0.85 using RF classifier; meanwhile, these were 0.794, 0.877, and 0.818 using SVM classifier. Additionally, we also confirmed the combination of RP11-138J23.1 and HuR in CLIP-seq data. To validate the physical interaction between RP11-138J23.1 and HuR, we carried out the RIP analysis and confirmed that RP11-138J23.1 could bind with HuR in BGC-823 cells (**Figure 6D**). Further analysis showed that HuR and RP11-138J23.1 cannot regulate each other’s expression (**Figures 6E, F**). Based on these observations, we propose that RP11-138J23.1 may function as an oncogene through binding HuR and exert regulation role at post-transcriptional level.

### VAV3 Is a Potential Downstream Target mRNA of “RP11-138J23.1-HuR”

In order to analyze the “RP11-138J23.1-HuR”-associated transcripts, we applied RNA sequencing and identified 852 genes being differentially expressed (fold-change >2, *p* < 0.05) after depletion of RP11-138J23.1, including 575 downregulated genes and 277 upregulated genes (**Figure 6G**). Gene Ontology (GO) and KEGG pathway analyses indicated that cell proliferation, cell growth, cell junction, and cell motility were involved in the affected functional processes in RP11-138J23.1-depeleted cells, which was consistent with the result of function assay (**Figure 6H**).

In a previous study, Xie et al. conducted high-solution transcriptome microarray in BGC-823 cells with HuR knockdown (13). We then explored potential targets that are regulated by “RP11-138J23.1-HuR” and found 139 genes (**Figure 6I**). Subsequently, qRT-PCR and Western blot assay validated a significant decrease of VAV3 observed after RP11-138J23.1 or HuR knockdown in BGC-823 and SGC-7901 cells (**Figures 6K, L**).

### RP11-138J23.1 and HuR Could Facilitate VAV3 mRNA Stability

**Figure 7:**
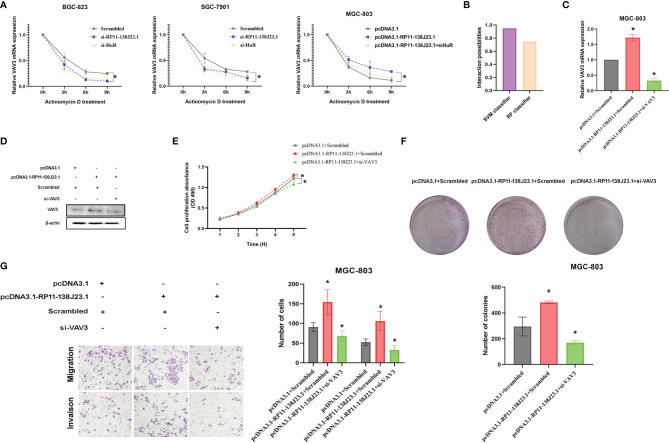
RP11-138J23.1 and HuR could increase VAV3 stability. **(A)** RNA stability assays were performed to measure degradation rates of VAV3 mRNA in BGC-823 and SGC-7901 cells with RP11-138J23.1 or HuR knockdown. In addition, RNA stability assays were also performed to measure degradation rates of VAV3 mRNA in MGC-803 cells with pcDNA3.1, pcDNA3.1-RP11-138J23.1, or pcDNA3.1-RP11-138J23.1+si HuR **(B)**. **(C)** The VAV3 mRNA expression level in MGC-803 cells transfect with pcDNA3.1+Scrambled, pcDNA3.1-RP11-138J23.1+Scrambled, and pcDNA3.1-RP11-138J23.1+si-VAV3. **(D)** The VAV3 protein expression level in MGC-803 cells transfect with pcDNA3.1+Scrambled, pcDNA3.1-RP11-138J23.1+Scrambled, and pcDNA3.1-RP11-138J23.1+si-VAV3. **(E)** MTT assays were used to measure the growth curve of pcDNA3.1+Scrambled, pcDNA3.1-RP11-138J23.1+Scrambled, and pcDNA3.1-RP11-138J23.1+si-VAV3-transfected MGC-803 cells. **(F)** Colony-forming assays were conducted to determine the proliferation of pcDNA3.1+Scrambled, pcDNA3.1-RP11-138J23.1+Scrambled, and pcDNA3.1-RP11-138J23.1+si-VAV3-transfected MGC-803 cells. **(G)** Transwell assays were used to determine the cell metastatic ability of pcDNA3.1+Scrambled, pcDNA3.1-RP11-138J23.1+Scrambled, and pcDNA3.1-RP11-138J23.1+si-VAV3-transfected MGC-803 cells. The data represent the mean ± SD from three independent experiments. ^*^ *p* < 0.05; ^**^ *p* < 0.01.

HuR is the ubiquitous member of a family of RBPs and involved in the regulation of mRNA stability (14). To better understand the impact of “RP11-138J23.1-HuR” on VAV3 mRNA stability, we treated BGC-823 and SGC-7901 cells with actinomycin D. Notably, qRT-PCR analysis showed that the half-life of VAV3 mRNA was significantly decreased in RP11-138J23.1- or HuR-downregulated GC cells (**Figure 7A**). Whereas, in RP11-138J23.1-overexpressed GC cells, VAV3 mRNA exhibited longer half-life (**Figure 7A**). Notably, the interaction probabilities of VAV3 mRNA with HuR were also predicted by RPISeq. The result showed 0.75 and 0.95 using RF classifier and SVM classifier, respectively (**Figure 7B**). The result of rescue assays also confirmed that RP11-138J23.1 exerts the mRNA stability-promoting effect depended on HuR protein. We found that the phenomenon of RP11-138J23.1-increased VAV3 mRNA half-life could eliminated by HuR knockdown.

### RP11-138J23.1 Promoted GC Cell Proliferation and Invasion Through HuR/VAV3 Pathway

The biological functions of VAV3 in GC cells were explored in the study of Xie et al., but whether VAV3 is involved in RP11-138J23.1-induced biology remains unclear. To determine whether RP11-138J23.1 exerts function *via* the HuR/VAV3 pathway, we conducted a rescue assay by manipulating RP11-138J23.1 and VAV3 expression levels (**Figures 7C, D**). The result from MTT, colony-formation, and Transwell assays showed that MGC-803 cells co-transfected with pcDNA3.1-RP11-138J23.1 and VAV3 siRNAs could partially reverse RP11-138J23.1-induced GC cell proliferation and metastasis (**Figures 7E–G**). Thus, we come to a conclusion that oncogenic activity of RP11-138J23.1 is partially attributable to its facilitation of VAV3 mRNA stability through association with HuR.

## Discussion

Recently, extensive efforts have revealed that lncRNAs involved in regulating the biological behavior of tumor cells by multiple molecular mechanisms, imply that they might serve as prognostic markers and therapeutic targets during cancer progression (15). LncRNA RP11-138J23.1, also named CRCAL‐3 or LINC02163:5, is located at chr5 (104079911-104105403). Through RNA-seq technology followed by TCGA dataset analysis, Yamada et al. originally discovered that RP11-138J23.1 contributed to colorectal carcinogenesis (16). Furthermore, *in vitro* experiments indicated the functional relevance of RP11-138J23.1 in association with cell cycle in colon cancer cells. Analogously, Tao and colleagues confirmed that RP11-138J23.1 upregulated colon cancer tissue and that the RP11-138J23.1 level was negatively correlated with patients’ overall survival and progression‐free survival (17). Notably, Wu and Yang found that m6A can regulate the expression of RP11-138J23.1 in CRC tissues. They also discovered that RP11-138J23.1 posttranslationally prevented the proteasomal degradation of Zeb1 *via* accelerating the mRNA degradation of Siah1 and Fbxo45 by binding to hnRNPA2B1 (18). These two independent pieces of work arrive at essentially the same conclusions that RP11-138J23.1 functioned as an oncogene in colon cancer. It is worth noting that LINC02163:6 and LINC02163:7 are also transcribed from the LINC02163 gene in addition to RP11-138J23.1. Meanwhile, several studies reported the critical carcinogenic functions of LINC02163 in different human cancers. For instance, LINC02163 was demonstrated to regulate colorectal cancer cell function by miR-511-3p/AKT3 axis (19). In another example, by serving as a competing endogenous RNA for miR-511-3p, LINC02163 upregulated the level of high mobility group A2 (HMGA2) and promoted the progression of breast cancer (20). More importantly, Wu et al. revealed that LINC02163 is involved in the growth and epithelial-to-mesenchymal transition phenotype of gastric cancer *via* miR-593-3p/FOXK1 axis (21). However, we detected that there was no relationship between RP11-138J23.1 and FOXK1 in our RNA sequencing data, which implied RP11-138J23.1 might exert carcinogenic effect through different molecular mechanisms. In the current study, we found that RP11-138J23.1 was upregulated in gastric cancer cell lines and tissues. As expected, high RP11-138J23.1 level was associated with poor prognosis in GC cases. RP11-138J23.1 knockdown impaired the ability of GC cell proliferation and metastasis *in vitro*. Furthermore, we reconfirm the effect of RP11-138J23.1 decrease on GC cell biological function in a subcutaneous xenograft model. On the whole, these results suggested that RP11-138J23.1 acted as an oncogene in GC development, which was similar with the findings in colon cancer.

It is well documented that lncRNAs can regulate genes expression at both the posttranscriptional and transcriptional levels (22). Although competing for microRNA binding is a crucial manner through which lncRNAs are involved in posttranscriptional gene regulation (23), lncRNAs are also involved in another layer of posttranscriptional processing *via* their modulation of mRNA half-life (24). HuR (ELAVL1), a member of the Elav family of RNA-binding proteins (RBP), is an established factor in RNA stabilization (25). It is known to bind to the AU-rich elements (AREs) that present at the target mRNAs’ 3′-UTR region (26, 27). An increasing evidence highlighted the importance of lncRNA-HuR complex in diverse conditions, especially in cancer (28, 29). Recent study has reported lncRNA HMS recruited HuR to stabilize the 3′-UTR of HOXC10 mRNA (30). In our previous study, we found that DUXAP10 can bind with HuR to maintain β-catenin mRNA stability and increase its protein levels (12). Here, we found that HuR could physically interact with RP11-138J23.1, thereby increasing the stability of VAV3 mRNA.

VAV3 gene encodes an ~25-kDa protein that is the activator of GTP kinase of Rho family (31). Previous study identified that VAV3 plays a crucial role in cell transformation by PI3K activation in NIH3T3 cells (32, 33). Subsequent to this discovery, VAV3 has been found to be overexpressed in various cancers, such as breast cancer, colorectal cancer, and gastric cancer (34–36). Highly expressed VAV3 can promote tumor cell growth, metastasis, and drug resistance. It was recently shown that the level of VAV3 in the peripheral blood could serve as a marker of predicting lymphatic metastasis for gastric cancer patients (37). Our present study further explored underlying molecular mechanisms of VAV3 mRNA upregulation in GC cells. However, the current study is limited by the relatively small clinical tissue sample size. Thus, the clinical value of RP11-138J23.1 may result in a selection bias. Moreover, whether RP11-138J23.1 directly interacts between VAV3 mRNA deserves further investigation.

In conclusion, the present study demonstrated that RP11-138J23.1 was upregulated in gastric cancer and associated with poor survival. RP11-138J23.1 exerts its oncogenic function by promoting cell proliferation and invasion through interacting with HuR protein and increasing the stability of VAV3 mRNA. These findings advance our understanding of posttranscriptional regulation of lncRNAs in GC progression.

## Data Availability Statement

The datasets presented in this study can be found in online repositories. The names of the repository/repositories and accession number(s) can be found in the article/**Supplementary Material**.

## Ethics Statement

The studies involving human participants were reviewed and approved by the Institutional Review Board of Outdo Biotech. The patients/participants provided their written informed consent to participate in this study. The animal study/experiment was reviewed and approved by the Institutional Animal Care and Use Committee of Zhejiang University.

## Author Contributions

JL and NL did the assays *in vitro* and collected clinical samples and analyzed the data. YX, XY, JX, and JWX performed experiments *in vitro* and *in vivo*. WL, JWX, and HJ wrote the manuscript. GY, SD, and FN designed this study. All authors have read and approved the final manuscript.

## Funding

The work was supported by grants from the National Natural Science Foundation of China (No. 81802309 to YX), Medical and Health Research Project of Zhejiang Province (No. 2019RC284 to YX), Young Talents Project of Huzhou Central Hospital (No. 2020YC10 to YX), and Project of Zhejiang Basic Public Benefit Research of Zhejiang Province (No. LGF18H160005 to GY).

## Conflict of Interest

The authors declare that the research was conducted in the absence of any commercial or financial relationships that could be construed as a potential conflict of interest.

The reviewers TX, PM declared a shared parent affiliation [Nanjing Medical University] with the authors JX, FN to the handling editor at time of review.

## Publisher’s Note

All claims expressed in this article are solely those of the authors and do not necessarily represent those of their affiliated organizations, or those of the publisher, the editors and the reviewers. Any product that may be evaluated in this article, or claim that may be made by its manufacturer, is not guaranteed or endorsed by the publisher.
